# Recycling Metchnikoff: Probiotics, the Intestinal Microbiome and the Quest for Long Life

**DOI:** 10.3389/fpubh.2013.00052

**Published:** 2013-11-13

**Authors:** Philip A. Mackowiak

**Affiliations:** ^1^Medical Care Clinical Center, VA Maryland Health Care System, Baltimore, MD, USA; ^2^Department of Medicine, University of Maryland School of Medicine, Baltimore, MD, USA

**Keywords:** Metchnikoff, microbiome, Lactobacilli, phagocytes, senility

## Abstract

Over a century ago, Elie Metchnikoff theorized that health could be enhanced and senility delayed by manipulating the intestinal microbiome with host-friendly bacteria found in yogurt. His theory flourished for a time, then drifted to the fringe of medical practice before re-emerging in the mid-1990s as a concept worthy of mainstream medical attention. Metchnikoff also predicted the existence of bacterial translocation and anticipated theories linking chronic inflammation with the pathogenesis of atherosclerosis and other disorders of the aged.

“The promise of microbiome research results largely on the future of probiotics…. Eventually, it may become possible to restore the health of a depleted microbiome simply by swallowing a capsule crammed with billions of bacterial cells, or by eating yogurt (1).”

Although Michael Specter implied otherwise in his article in *The New Yorker* (1), neither microbiome research nor the use of probiotics to promote health is new. Over a century ago, Ilya Ilyich (Élie) Metchnikoff (Figure 1) theorized that health could be enhanced, and also that senility could be delayed, by manipulating the intestinal microbiome with host-friendly bacteria found in yogurt (2). His theory flourished for a time, then drifted to the fringe of medical practice, only to re-emerge in the mid-1990s as a concept worthy of mainstream medical attention. Today probiotics are not only the subject of intense medical research but also the source of a multi-billion dollar global industry (3). The microbiome now has a N.I.H.-funded Human-Microbiome Project^1^, as well as a new peer-reviewed journal devoted to it exclusively^2^.

**Figure 1:**
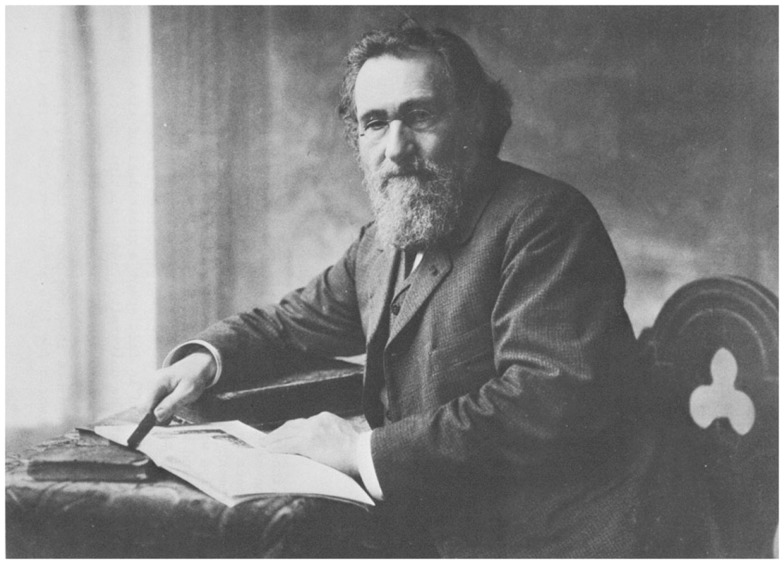
**Elie Metchnikoff (1845–1916)**.

Metchnikoff’s concepts laid the foundation for fecal transplantation, predicted the existence of bacterial translocation from the intestinal lumen into the bloodstream and lymphatic system, and anticipated theories linking chronic inflammation with the pathogenesis of atherosclerosis (2). His concepts have also been invoked in modern times by fringe groups, such as the medical ecologists, attributing a host of chronic disabilities to intestinal proliferation of *Candida* sp. (4).

Elie Metchnikoff was born on May 3, 1845 near Kharkow, Russia (now part of Ukraine). He studied at the university there from 1862 to 1864 before traveling to Germany where he trained in comparative anatomy at universities in Giessen, Gottingen and Munich. In 1870 he accepted a professorship at the University of Odessa, worked there for 12 years, and then resigned his post to begin an odyssey that took him from Madeira to Teneriffe to the Volga. It was during these wandering years that he published his opus magnum, “Intracellular Digestion in Invertebrate Animals,” in *Arberten* of the Zoological Institute of Vienna. In it he summarized studies demonstrating clearance of foreign particles from the tissues of lower animals by devouring cells he called “phagocytes,” which he came to realize were critical in protecting the host animal against infection. The work earned him an appointment as Pasteur’s successor as Director of Scientific Research at the Pasteur Institute, a Nobel Prize in 1908, and immortality as the father of cellular immunology (2).

Late in his career, as his vigor began to ebb, Metchnikoff turned his attention to the mechanisms responsible for senility and the means by which they might be inhibited. His ideas concerning these issues were dominated by both his work with phagocytes and his own ascetic life style (2).

In contemplating questions related to senility, Metchnikoff’s attention was drawn to the residents of Eastern Europe, in particular those of the Balkan States and Russia, among whom there existed an unusually large number of centenarians. He was struck by the fact that citizens of that region who lived to the age of 100 were, for the most part, poor or of humble circumstances with extremely simple life styles. This convinced him that the secrets to a long, productive life included a simple, sober existence, “moderate in food and drink and in all other pleasures,” complimented by daily exercised “whatever be the weather… lived in a peaceful environment in which the air is pure… going to bed early and rising early after not more than 6–7 h of sleep, bathing daily with water neither too hot nor too cold, engaging in regular work and mental stimulation, and avoiding alcohol, other stimulants and narcotics (2).” Few today would argue with any aspect of Metchnikoff’s concept of a healthy life style, with the possible exception of his proscription against alcohol, given the current excitement over the possibility that red wine might be cardio-protective (5).

Macrophages and the colonic microbiome, however, were the focus of Metchnikoff’s most innovative thinking with regard to mechanisms responsible for senility. He believed that virtually all of the disabilities of old age are the work of phagocytes transformed from defenders against infection into destroyers of healthy tissues by *autotoxins* derived from putrefactive bacteria residing in the colon. According to his concept, senile degeneration of the nervous system, for example, is the work of *neurophags* induced by autotoxins to devour the contents of nerve cells and cause them to atrophy; the blanching of aging hair, the work of similarly activated *chromophags*; muscle atrophy, the work of *sarcophags*; osteoporosis, the work of *osteophags* (osteoclasts), and so on. Such degenerative changes, he believed were nearly always premature and potentially prevented by measures directed against the colon’s putrefactive bacteria (2).

Metchnikoff dismissed the human colon as a “vestigial cesspool” that functions as little more than a reservoir for waste matter in which microbes produce “fermentations and putrefaction harmful to the host.” Production of autotoxins by the colonic microbiome, he believed, is amplified by the western diet, and that avoiding alcohol and foods “such as rich meats and raw or badly cooked substances containing microbes” might diminish colonic bacterial production of the noxious ptomaines (alkaloids) responsible for premature senility. Others tried unsuccessfully to completely eradicate the colonic microbiome and its autotoxins by removing the colon or by administering antibacterial enemas. Metchnikoff advised against such measures. He believed that the evils of autointoxication are best mitigated by inhibiting the putrefactive bacteria in the colon with lactate-producing bacteria (2).

The specific regimen recommended by Metchnikoff for suppressing putrefactive colonic bacteria consisted of daily doses of *probiotics* in the form of “soured milk (i.e., yogurt) prepared by a group of lactic bacteria, or of pure cultures of the *Bulgarian bacillus* (*Lactobacillus bulgaricus*), but in each case (accompanied by) a certain quantity of milk, sugar, or sucrose.” He followed his own advice as a regular part of his diet and was “very well pleased with the result.” What, exactly, he meant by “very pleased with the results” is not clear. Possibly, he was encouraged by a diminution of “sulfo-conjugate ethers” in his urine, which were then monitored as surrogate markers for intestinal putrefaction. Several of his friends who “suffered from maladies of the intestine or kidneys” followed his example and, likewise, were “well satisfied” with the results. The efficacy of his treatment in retarding the onset of senility was never validated scientifically (2). Moreover, subsequent work by a host of investigators showed that *L. bulgaricus* is unable to survive in the human intestine or that of any other animal studied and, hence, not capable of replacing putrefactive bacteria in the colon (6). *L. acidophilus*, however, can live in the human intestine and has been used with limited success as a probiotic.

The FAO/WHO (Food and Agriculture Organization of the United Nations and the World health Organization) defines *probiotics* as “live microorganisms which when administered in adequate amounts confer a health benefit on the host (7).” As noted above, interest in probiotics such as lactobacilli has soared in recent years, with many studies purporting to demonstrate their efficacy in alleviating human disease, and many more testimonials extolling their benefits. Nevertheless, according to the Cochrane Summaries^3^, evidence supporting such claims is limited to just a few conditions. Probiotics have been shown to be effective or possibly effective in preventing pediatric antibiotic-associated diarrhea, necrotizing enterocolitis in preterm infants, and upper respiratory tract infections. They have been shown to be effective or possibly effective in treating acute infectious diarrhea and persistent diarrhea in children. To date, probiotics, such as *L. acidophilus*, have not been shown to be effective against bacterial vaginosis, ulcerative colitis, Crohn’s disease, or preterm labor (2).

Fecal transplantation is a special form of probiotic therapy in which a healthy donor’s fecal microbiota in its entirety (i.e., processed stool) is transplanted into the intestine of a patient suffering with one of various intestinal disorders. The procedure was first performed in 1958 in patients with fulminate pseudomembranous enterocolitis (8). The results were immediate and spectacularly successful. Today patients with the same disorder – now known as “*Clostridium difficile* enterocolitis” – respond favorably to such therapy over 90% of the time. The remarkable success of fecal transplantation in such patients has raised hope that it might also be used to alleviate or prevent other intestinal disorders, such as inflammatory bowel disease, irritable bowel disease, colorectal cancer, and certain nutritional and metabolic disorders (9). However, to date, evidence supporting the efficacy of fecal transplantation against any of these disorders is lacking.

Metchnikoff’s recommended use of a probiotic to delay the onset of senility, while never embraced by mainstream medicine, has been resurrected by fringe elements like *Body Ecology*, which promote a “back to basics approach to restoring health (10).” They claim that probiotics help one “age gracefully,” without revealing the mechanism by which such benefit might be brought about.

Metchnikoff was not a physician. However, he believed as physicians do today, that death is rarely if ever “natural,” but in nearly all cases is the result of either an accident or some treatable or potentially preventable disease. He maintained that if these can be avoided, one might expect to survive the full extent of the human life cycle (*orthobiosis*) before at last succumbing to “natural death.” The number of years actually comprising the normal human life cycle has long been a source of speculation and debate. In the United States today, the life expectancy for men is 75.9 years, and for women 80.7 years, averages which have risen steadily for many decades (11). But do they represent “orthobiosis” – the maximum number of years a man or a woman can expect to survive if not killed by an accident or disease? Metchnikoff thought not. He believed that the normal life cycle of humans is 100 years. Some have speculated that the cycle might be as long as 200 years, given rare reports of persons reaching the ages of 130, 150, and even 185 years of age (2). Whatever the upper limit of the normal life cycle of humans, Metchnikoff was convinced that a person could enhance the chances of reaching it by simple, sober living, and daily consumption of “soured milk” beginning in childhood and continued thereafter. Personally, he fell well short of orthobiosis as he envisioned it, dying at age 71 of heart failure (12).

If Metchnikoff’s regimen could increase the average life expectancy, would that be a good thing? The U.S. government, like governments of other countries, is already hard-pressed to meet the medical needs of its elderly citizens. If old people were to live longer, wouldn’t they consume even more of the resources needed to support the population at large? Metchnikoff brushed aside such concerns, maintaining that if the causes of precocious senility were eliminated, it would no longer be necessary to pension persons at 60 or 70 years of age. If science allowed them to complete their normal life cycle, given their vast experience, old persons might benefit rather than burden society by fulfilling their role as society’s wisest advisers and judges. If old age could be “postponed so much that men of from 60 to 70 years of age (were to) retain their vigor… young men of 21 years of age (would) no longer be thought mature or ready to fulfill functions so difficult as taking a share in public affairs (2).”

The average life expectancy in France during Metchnikoff’s day was not much more than 40 years (13). Today in the United States it is nearly twice that number, as are the number of elderly people supported by government programs. Whether it would be good to increase the average life expectancy of Americans further is a question asked today only in relation to the cost of end-of-life care. Although death is inevitable, Americans – physicians as well as laypersons – nearly always view it as unnatural. Thus, like Metchnikoff, Americans believe that extending life, in particular, useful, productive, healthy life, is a good thing. Metchnikoff’s formula for doing so is simple, inexpensive, and logical. Unfortunately, there is little reason to believe that it is capable of producing the optimistic results he predicted it could.

## Conflict of Interest Statement

The author declares that the research was conducted in the absence of any commercial or financial relationships that could be construed as a potential conflict of interest.

## Footnotes

^1^http://www.human-microbiome.org/

^2^http://www.eurekalert.org/pub_releases/2013-01/uod-gsl010913.php

^3^http://summaries.cochrane.org/search/site/probiotics/systematic
